# Time to diagnosis of Duchenne muscular dystrophy in Austria and Germany

**DOI:** 10.1038/s41598-022-27289-2

**Published:** 2023-01-05

**Authors:** Miriam Hiebeler, Simone Thiele, Peter Reilich, Günther Bernert, Maggie C. Walter

**Affiliations:** 1 Department of Neurology, Friedrich-Baur-Institute, Ludwig-Maximilians-University of Munich, Ziemssenstr. 1, 80336 Munich, Germany; 2 Department of Pediatrics, Klinik Favoriten, Vienna, Austria

**Keywords:** Neuromuscular disease, Paediatric neurological disorders

## Abstract

Duchenne muscular dystrophy (DMD) is an X-linked genetic disorder manifesting in early childhood with progressive muscular weakness and atrophy, and resulting in early loss of ambulation. The collection and evaluation of epidemiological data for this disease is crucial for an early diagnosis and disease management. In Germany, data are collected via the TREAT-NMD DMD patient registry (www.dmd-register.de). In contrast, data collection in Austria has not yet been performed systematically. For collecting data from Austrian DMD patients, an online survey of the patient’s caregivers was conducted. Data of 57 patients were collected entailing initial symptoms, diagnosis and therapeutic measures. Comparable data has been collected for Germany via the TREAT-NMD DMD patient registry. 57 DMD patients aged 4–34 years completed the Austrian survey. On average, first symptoms of the disease appeared at the age of 3.1 years. As the most frequent first symptom, 46% of the patients described problems in climbing stairs. In 40% of the patients, DMD was diagnosed early due to an accidentally detected hyperCKemia in infancy or early childhood. Corticosteroids represented the main therapeutic option in our cohort. At the time of the survey, only 52% of the patients were treated with corticosteroids. Patients from Germany reported that first symptoms appeared at the age of 3.06 years. Diagnosis was established by genetic testing or muscle biopsy. 47% of the patients were treated with corticosteroids. Time between first symptoms and diagnosis was 7 months in Austria, and 4.7 months in Germany, respectively. Compared to earlier international studies, the Austrian data show encouraging results regarding earlier start of corticosteroid therapy in a larger percentage of patients. Austrian and German data show a trend towards an earlier diagnosis of DMD, while the age at symptom onset was similar to previous studies. The collection and evaluation of epidemiological data of DMD patients is important and will hopefully contribute to accelerate DMD diagnosis and treatment access for the patients.

## Introduction

Duchenne muscular dystrophy (DMD) is an X-linked genetic disorder manifesting in early childhood with progressive muscle weakness resulting in loss of ambulation. Following loss of ambulation, cardiac and respiratory symptoms occur, leading to premature death^1,2^. With an incidence of 1: 3600–6000 of male newborns, DMD is one of the most frequent neuromuscular diseases^1,3^. First symptoms in form of muscle weakness usually appear around age three; however, more unspecific signs of developmental delay can be detected earlier^1,4^, or an accidentally found hyperCKemia leads to a presymptomatic diagnosis. Diagnosis is confirmed by genetic testing^5^. Still, mean patient age at diagnosis in Germany is 3.8 ± 2.4 years, and mean time from report of first symptoms to diagnosis is reported with 1.4 ± 1.8 years^6^.

Patient registries in rare diseases are an essential tool for research. Within TREAT-NMD ("Translational Research in Europe for the Assessment and Treatment of Neuromuscular Diseases"), a "Network of Excellence" funded by the European Union, national patient registries for DMD with a harmonized mandatory dataset have been established throughout Europe since 2007.

The German DMD patient registry is open for Austrian patients; however, not many patients from Austria have registered. To overcome this data lack, a survey for Austrian DMD patients and their families has been conducted recently. The survey aimed at promoting an early diagnosis of DMD and identifying approaches to enable patient’s access to treatment and standards of care.

Here we present the results of the Austrian survey in comparison with the German TREAT-NMD DMD patient registry data, and evaluate these findings within the published data from other countries.

## Methods and cohort

Data from Austria were collected by an online survey and distributed via the Austrian Muscle Research Organization (ÖMF) and the Marathon Association (Association of parents and relatives against muscle diseases in children). Parents or relatives of patients as well as members of the Marathon Association were informed via regular mail about the survey and asked to participate. The information letter was sent to 200 potential participants and contained a link to the online survey; data was processed anonymously. All methods were performed in accordance with the relevant guidelines and regulations. We confirm that informed consent was obtained from all subjects and/or their legal guardians.

In total, 57 Austrian patients/caregivers completed the questionnaire; data on 57 male DMD patients aged 4–34 years were collected with the following age distribution: 17% of the patients up to age 5, 49% between age 6 and 18, and 36% between age 19 and 34.

The questionnaire asked for details on living arrangements, educational degree and profession, initial symptoms and age at symptom onset, age at diagnosis, diagnostic methods applied (including results of genetic testing), aids (assistive devices) and specific treatment (corticosteroids). Utilization of multidisciplinary approaches, e.g. psychotherapy and respiratory support, were also requested, and the level of caregiver’s stress was assessed by five questions, each of which allowed respondents to indicate their stress level on a scale of 1–5 (see Online Appendix S1).

When the Austrian survey was performed, 766 German participants with genetically identified DMD between age 0–45 were registered in the TREAT-NMD patient registry (www.dmd-register.de). The registry is patient-based; the patients or their caregivers register themselves and send a copy of the genetic report to the registry headquarters at the Friedrich-Baur-Institute, Dept. of Neurology, Ludwig-Maximilians-University of Munich. Patient’s data are annually revisited and updated.

Within the registered patients, 7% were younger than 5 years, 53% between 6 and 18 years, and 40% between 19 and 45 years. However, not each item of the Austrian questionnaire was available for the individual patients from the German TREAT-NMD patient registry. Therefore, only corresponding data were utilized for matching the Austrian survey results with the TREAT-NMD registry information and for highlighting the results within an international context.

Descriptive statistics were performed, and significant differences were calculated by Pearson's chi-square test using the SPSS 26 software. A value of p < 0.05 was considered as statistically significant.

### Ethics approval

Ethics approval for Austria was granted by the magistrate of Vienna in May 2019; for utilization of the German DMD patient registry, ethics approval had been granted by the ethics board of the Ludwig-Maximilians-University of Munich, Germany since September 2007. All methods were performed in accordance with the relevant guidelines and regulations. We confirm that informed consent was obtained from all subjects and/or their legal guardians.

## Results

### Austrian survey

The Austrian survey was completed by 57 patients between 4 and 34 years, out of approximately 200 known DMD patients in Austria.

The majority (93%) of the patients lived with their parents, the remaining 7% with other related caregivers, e.g. siblings or grandparents. 49% of patients were still attending school, 2% were currently in apprenticeship training, 2% were seeking employment, while 5% had current employment and another 5% were attending university.

**Figure 1:**
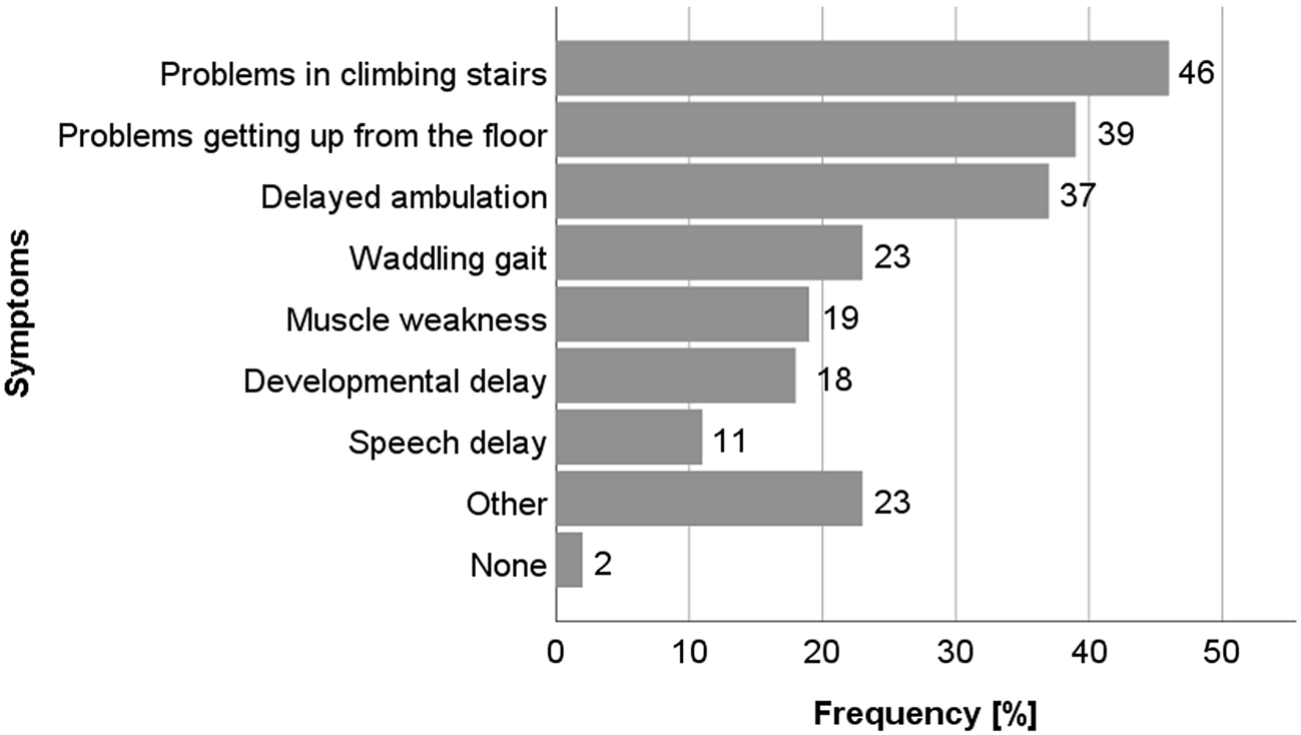
First symptoms of Austrian patients.

Figure 1 shows the first symptoms reported by the patients. On average, first symptoms of the disease appeared at the age of 3 years, ranging between 9 months and 7 years.

Most frequently (in 46% of patients), problems in climbing stairs were reported as a first symptom, followed by problems in getting up from the floor (39%) and delayed ambulation (37%). Patients additionally reported speech delay, waddling gait, and loss of strength. In 40% of the patients, DMD was only diagnosed due to accidentally detected hyperCKemia in early infancy. 60% of patients up to age three first visited a pediatrician, while patients > 3 years directly went to a neuropediatrician (52% of patients). Mainly, DMD was diagnosed in specialized neuromuscular centers (75% of patients).

**Figure 2:**
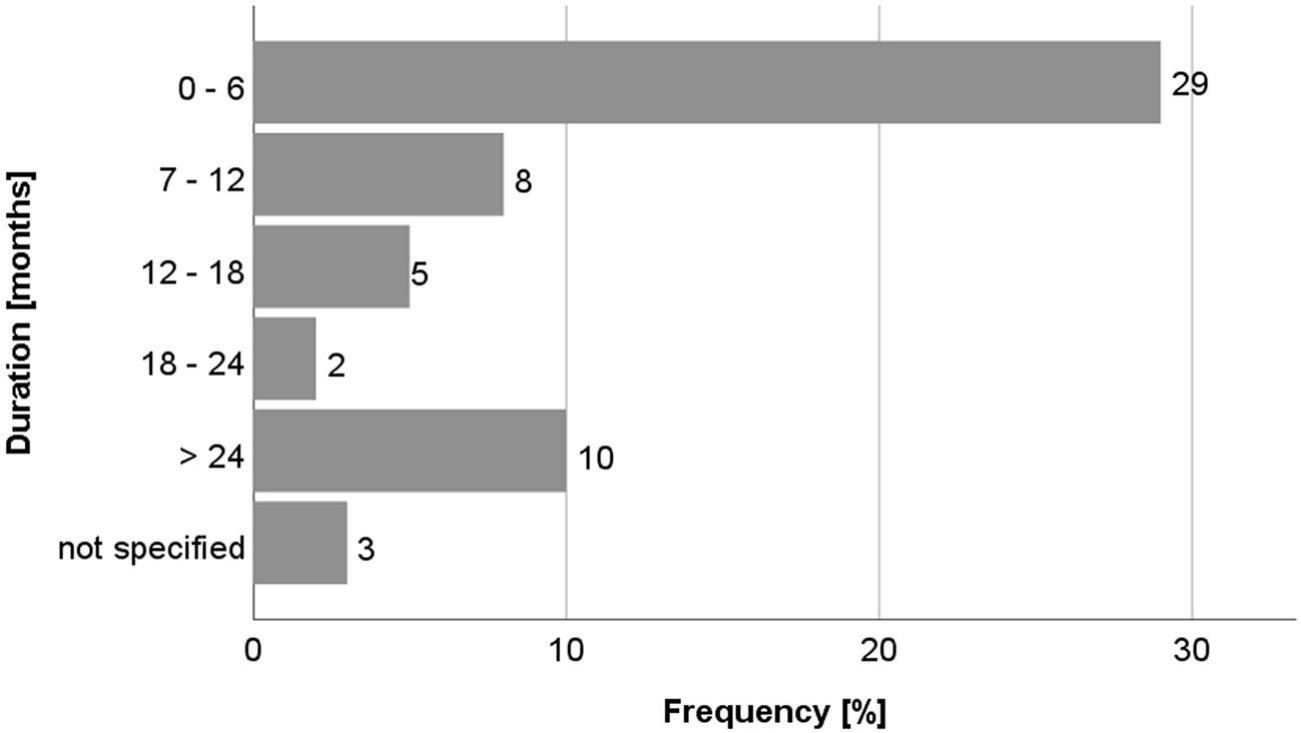
Time to diagnosis in Austrian patients.

**Figure 3:**
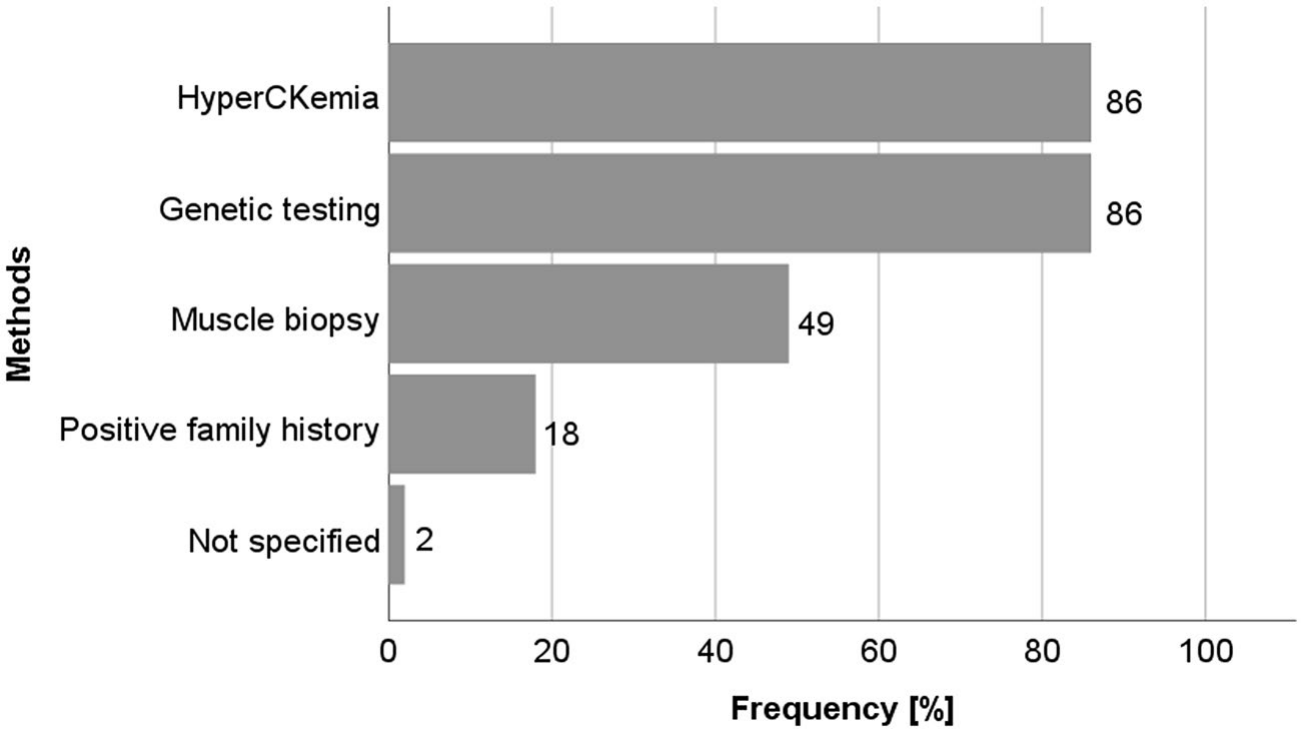
Diagnostic methods in Austria.

Figure 2 shows the delay between first symptoms and final diagnosis. In half of the patients (51%), duration between first symptoms and final diagnosis was only six months; however, in one out of five patients (18%), the diagnostic process took over two years. In 86% of patients, hyperCKemia was leading to DMD diagnosis. Interestingly, CK levels were predominantly (42%) analyzed in patients who had been diagnosed with DMD (diagnosis known for 6 months or less). In only 86% of the patients, genetic testing was performed; the rest was histologically diagnosed by muscle biopsy, or clinically via a positive family history (Fig. 3).

Muscle biopsy was predominantly performed in older patients (26% up to age 10, 63% age 11–20, and 71% age 21–34). Differences in the distribution between the age groups were statistically significant (p = 0.01).

In patients who had genetic testing, diagnosis of DMD was established at a mean age of 3.7 years (median 3.5 years), ranging from 2 months to 9 years.

**Figure 4:**
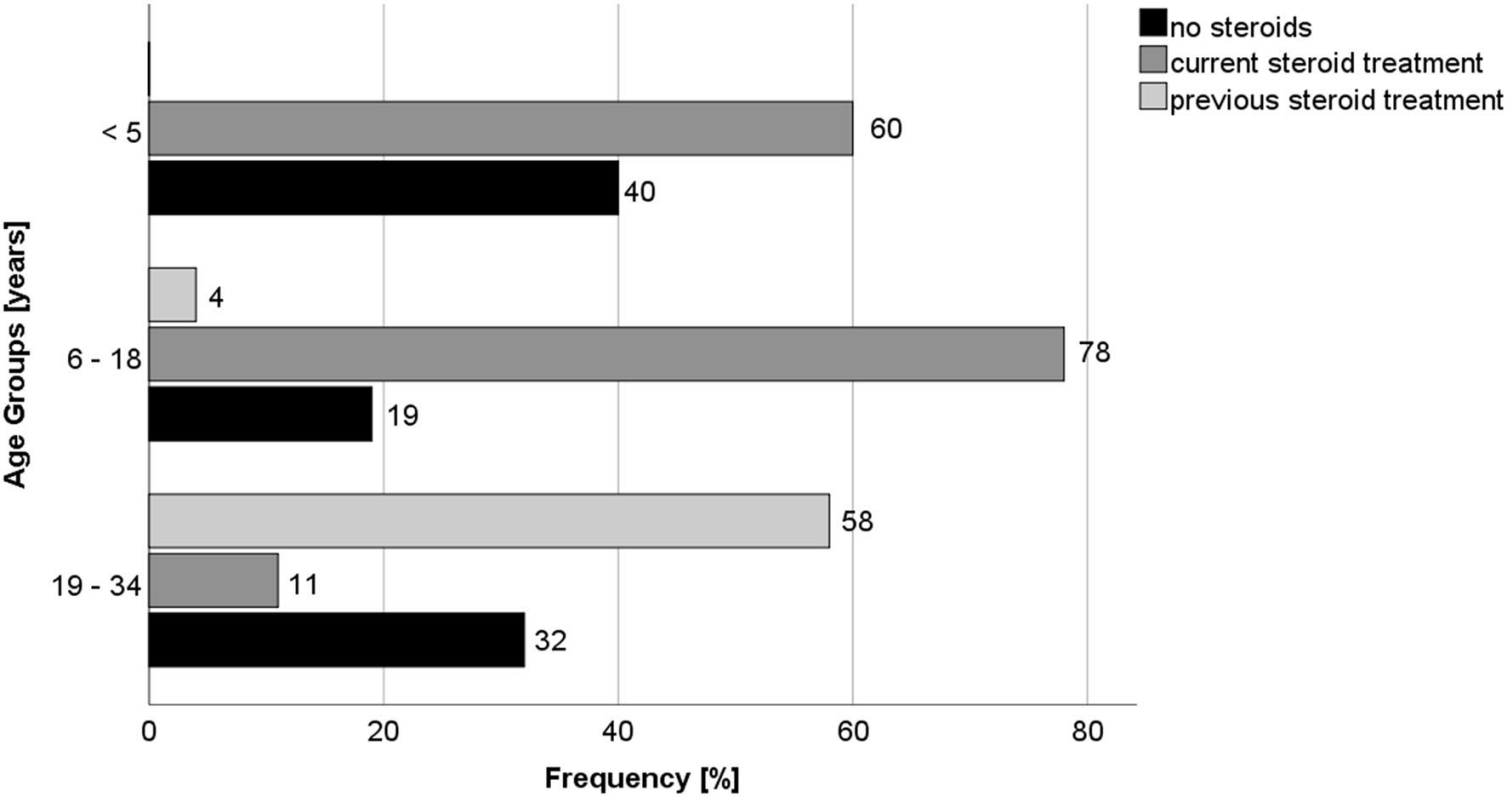
Corticosteroid therapy by age groups (Austria).

Treatment with corticosteroids constituted the main therapeutic option. At the time of the survey, 52% of all patients were treated with corticosteroids, mainly in 6- to 18-year-old patients (78%); in the group of 19 to 34-year-old patients, 58% had received steroids in the past, and 11% were still treated (Fig. 4). The differences of corticosteroid treatment in the respective age groups proved to be statistically significant (p < 0.001).

Start of steroid treatment ranged between 3.4 and 11 years of age. Half of the patients started corticosteroid therapy at the age of 5.5 years.

6/16 patients (37.5%) without prior corticosteroid therapy were still able to walk (ambulatory) at the time of the survey. In patients already treated with corticosteroids, 21 of 40 patients (52.5%) were still ambulatory. Unfortunately, mean age and age range of the patients were not statistically calculated due to the small number of patients.

Patients lost ambulation at a mean age of 10.3 years (ranging from 5 to 16 years; median 10 years). These patients were completely wheelchair dependent. In the age group from 19 to 34 years, all patients had lost ambulation.

During the course of the disease, patients needed wheelchairs (60%), ortheses (37%) and standing aids (16%). Among ambulatory patients, ortheses were the most commonly used assistive device (41% of this group). 44% of ambulatory patients did not use any kind of assistive device.

72% of the non-ambulatory patients had orthopedic surgery, most frequently pes equinus correction, followed by scoliosis operation.

Data regarding ventilation could only be collected for 9 patients. Non-invasive ventilation was needed in 78% of these patients (n = 7), and invasive ventilation in the remainder (n = 2). Psychotherapeutic support was provided to 29% of patients, predominantly in non-ambulant patients up to age 18 (50%).

For assessment of care, patients were asked if care was provided by the family or external help services; results were correlated with the stress level of the caregiver, which had also been assessed by a questionnaire. In 94% of all patients, the family provided the required care; 28% additionally used mobile nursing services. 12% of the patients used 12- to 24-h care services. Caregivers who provided full-time care for the patients were mainly burdened by the physical strain of the home care.

### German registry ("Treat-NMD/DMD")

The German DMD patient registry (www.dmd-register.de) includes 766 patients with genetically confirmed DMD, aged 0–45 years. Unfortunately, not all data was available for each patient. Patient registry data are frequently incomplete due to lack of follow-up thus enabling easy collection and processing. The amount of evaluated patients is indicated, respectively.

In this cohort, mean age at first symptoms was 3 years (ranging from 3 months to 12.92 years; n = 103). Mean age of diagnosis was 3.5 years (ranging from 0 to 12 years; n = 160). 80% (n = 128) of the 160 patients were identified by genetic testing, 15 patients received an additional muscle biopsy. However, only 20% (n = 32) were diagnosed by muscle biopsy alone. Comparing mean age at symptom onset with mean age at diagnosis results in mean 4.7 months of time to diagnosis in Germany. However, data for age at symptom onset and age at diagnosis was only available for 103 out of 160 patients.

Data on corticosteroid treatment were available for 204 patients.

**Figure 5:**
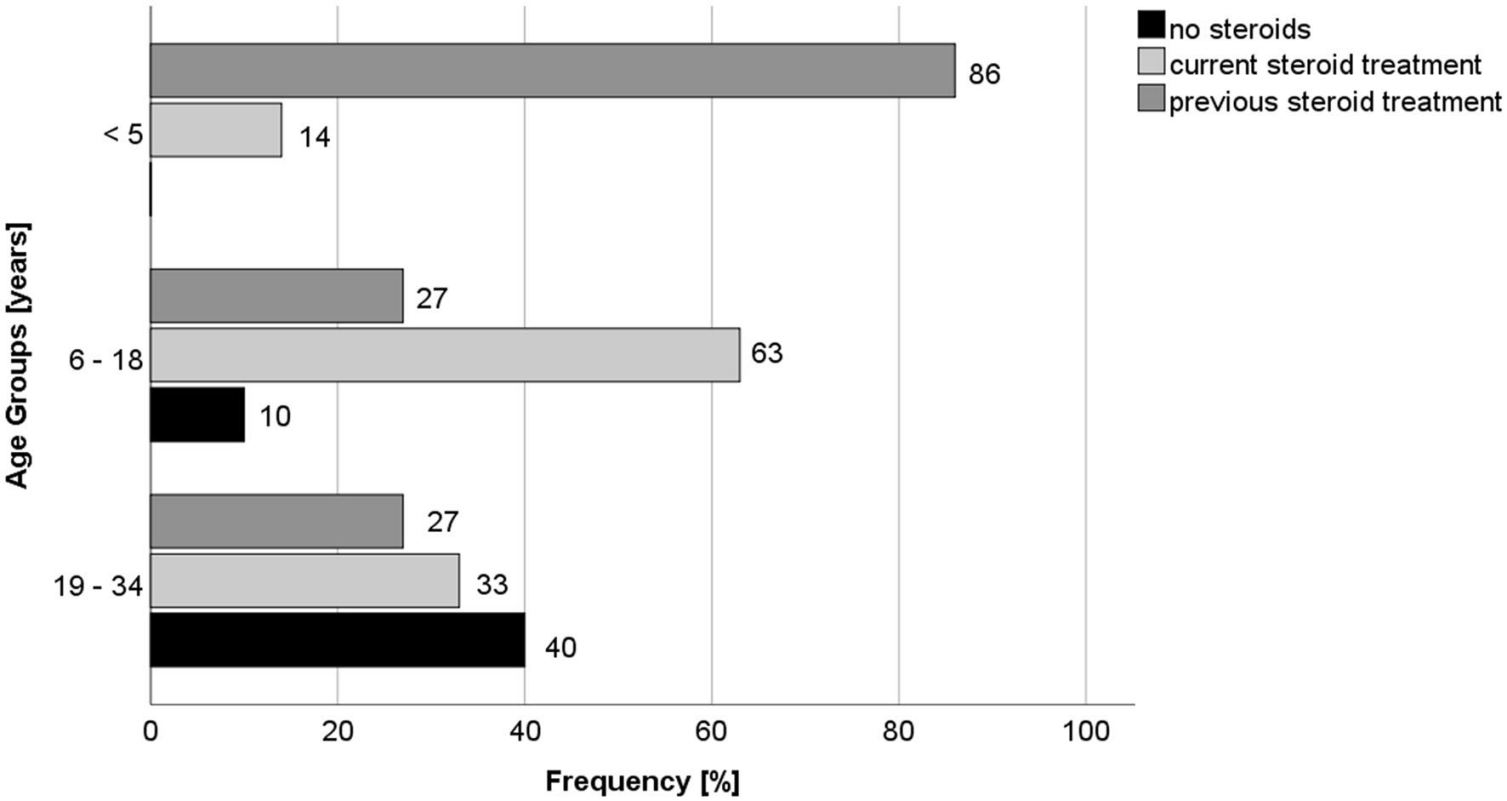
Corticosteroid therapy by age groups (Germany).

47% of the patients were currently treated with corticosteroids; another 16% had been treated in the past. Predominantly, patients aged 6–18 were treated (63%), whereas only 14% of patients below age 5 received steroids (Fig. 5). Interestingly, in Austria 60% of patients below age 5 already received corticosteroids. Statistical analysis shows a significant difference regarding corticosteroid treatment between Germany and Austria in the group of young DMD patients below age 5 (p = 0.003), while differences in the remaining age groups were not significant.

Data on ambulation were available for 198 patients. The majority of patients (89%) up to age 5 were ambulant. In patients between 6 and 18 years, 68% were still able to walk. In patients aged 19 and older, all patients had lost ambulation.

For age at loss of ambulation, data from 45 patients could be evaluated. Mean age at loss of ambulation was 11.5 years (ranging from 12 to 17.7 years).

## Discussion

Timely diagnosis of DMD continues to be a challenge for the treating physicians. Data collection on Duchenne patients in different countries is important to improve knowledge of the disease and enable a more rapid diagnosis, leading to earlier treatment and thereby delay of disease progression^3^. For the first time, socioeconomic data on Austrian DMD patients were systematically collected, and compared to data from the German TREAT-NMD DMD patient registry.

Data on time to diagnosis and management in DMD cohorts have been published for other countries: Italy^7^, United Kingdom^5^, USA^8^, Australia^9^ and pan-European^6^. However, some overlap between patient populations^5,6^ and TREAT-NMD registry data can be presumed, since harmonized European registry data were utilized for these.

Mean age at symptom onset over all countries including Austria and Germany was 2.9 years. First symptoms were observed latest in Austria and Germany at about 3 years, compared with 2.6 years in Italy, 2.7 years in the UK and in the USA and 2.85 years in Australia. In Austria, DMD was diagnosed at a mean age of 3.7 years, 7 months after symptom onset. Diagnosis of DMD in Germany, Italy, Australia, UK, and USA was determined at 3.4, 3.4, 3.8, 4.3 and 4.9 years, respectively. Time to diagnosis was fastest Germany with 4.7 months; Italy required 10 months and Australia 11.5 months for DMD diagnosis, while the UK (19.2 months) and the USA (26.4 months) showed the longest delay until DMD was finally diagnosed^5,7–9^. Interestingly, the interval between symptom onset and diagnosis had not been changed within the past three decades in the US^5,7,8^. A cross-European comparison by Vry et al.^6^ detected a mean time to diagnosis of about 15 months, with a final diagnosis at a mean age of 4.3 years. Table 1 summarizes all data regarding time to diagnosis for the respective countries.

**Table 1:** Comparison of the diagnostic delay in different countries.

	Treat-NMD/DMD (Germany)	Austria	D’Amico et al. (Italy)	Wong et al. (Australia)	Vry et al. (*)	van Ruiten et al. (UK)	Thomas et al. (USA)
Mean age at first symptoms [mo]	36.7	37.0	31.0	32.5	36.0	32.5	32.4
Mean age at diagnosis [mo]	41.4	44.0	41.0	44.0	51.0	51.7	58.8
Time to diagnosis [mo]	**4.7**	**7.00**	**10.0**	**11.5**	**15.0**	**19.2**	**26.4**

Significant values are given in bold.

*Bulgaria, Czech Republic, Denmark, Hungary, Poland, Germany and UK.

Interestingly, countries with the least delay between symptom onset and diagnosis—Germany and Austria—were so far not able to detect first symptoms earlier, which would not only be crucial for an earlier diagnosis and timely treatment, but also for genetic counselling, implementation of standards of care, and participation in clinical trials.

In 1999, the mean age at diagnosis was 4.8 years in the UK^10^. In Austria, diagnosis is currently established at a mean patient age of 3.7 years, while in Germany patients are even younger with a mean age of 3.5 years at diagnosis. Compared to 1999, DMD is also diagnosed earlier in UK, Italy, Australia and across Europe (4.2 years on average for those countries), but not in the US^5,7–9^. A possible factor for the improvement in diagnosis age in Europe and Australia is more frequent laboratory testing of the transaminases and CK, mainly within the scope of routine examinations^7^, and accessibility to and reimbursement of genetic testing. This is in contrast to the USA, where geographic size limits access to diagnostic procedures. Overall, the delay in diagnosis in the USA results most probably from a complex mix of different factors, in addition to geographic size, e.g. the large difference in urban and rural medical care, lack of knowledge of primary care providers about DMD resulting in less CK and transaminase testing, and to a lesser extent today, insurance coverage issues, especially for genetic testing procedures.

In case of hyperCKemia, patients today are quickly referred to a neuropediatrician. In Austria, 75% of the patients were diagnosed by a neuropediatrician in a specialized center. Nevertheless, diagnosis as early as possible would be desirable, especially with regard to possible new therapeutic options, which should be started while muscle degeneration is still mild^3^.

Therefore, children’s routine check-ups in Germany (U7 between 21 and 24 months) and Austria (6^th^ MKP between 22 and 26 months) would be a good timepoint to raise awareness of DMD diagnosis, by looking out for unspecific developmental delay such as impaired cognitive, verbal and motor function in boys, elevated transaminases and/or HyperCKemia^3^.

At this point, it should be noted that co-occuring neurocognitive conditions seem to prolong the time to diagnosis. A recent work by Lee et al. shows a delay in diagnosis of about 1.5 years in patients with concomitant neurocognitive diseases, which were present in about 73% of patients with a diagnosis at age 5 years or later^11^.

Furthermore, additional factors may influence age at diagnosis. An US study by Counterman et al.^12^ found that socioeconomic factors such as insurance status, ethnic origin, or growing up with single parents influenced age at diagnosis. In addition, age at diagnosis is also different for specific DMD genotypes. In line with Vry et al.^6^, Counterman et al.^12^ identified diagnosis at a younger age within recent years, probably resulting from improved awareness of DMD symptoms and improved genetic testing methods^12^.

To date, regardless of the underlying specific mutation, treatment with corticosteroids in addition to physiotherapy is the gold standard of treatment in DMD, prolonging ambulation, and slowing down the decline of heart and lung function^2,13–15^. Additionally, mutation-specific therapies are available for patients with nonsense-stop mutations (approved in the EU from age 2) or particular exon deletions (approved in the US)^16^. Importantly, a successful therapy would have to start early in development and progression of the disease—preferably between age 2 and 3—to prevent the progressive loss of muscle and motor function^3,16^.

In Austria, corticosteroid therapy is started early (mean age at start of therapy 5.5 years). In comparison, Vry et al. indicate a later onset at 6.1 years throughout Europe^6^. As mentioned above, 78% of Austrian patients aged 6–18 years are currently treated with corticosteroids, while 19% never received them. In contrast, only 63% of the patients in Germany are treated with corticosteroids in the same age group, while 27% never even took steroids. Furthermore, in Austria 60% of patients up to 5 years of age are already treated with corticosteroids, whereas in Germany significantly less—only 14%—are treated likewise in the same age group. According to recent guidelines, it is recommended to start corticosteroid therapy at the age of 4–6 years, when muscle strength starts to decline^13,14^. In the highest age group of 19–35 years, 73% are currently or have previously been treated with corticosteroids, in Germany only 63%, respectively. However, in Germany more patients (33%) are still using corticosteroids in the oldest age group (11% in Austria, respectively). In summary, corticosteroid therapy is used earlier and more frequently in Austria than in Germany, corresponding to the recommendations of the current literature^13,14^. It remains unclear why corticosteroids are used later in live and overall, less frequently in Germany compared to other countries; an awareness campaign in cooperation with patient organizations would be warranted to improve steroid use and thereby standard of care for the German patients.

Regarding the differences in corticosteroid use, there are interesting differences between patients in both countries for the respective age groups. The group of < 5-year-old patients and the 6- to 18-year-olds are similar (< 5 years: 90% Austria, 89% Germany; 6–18 years: 63% Austria, 68% Germany). As expected, none of the patients are ambulatory in Austria and Germany in the oldest age group, respectively. However, despite later and less frequent use of corticosteroids in Germany, ambulation seems to be maintained equally long in both countries. However, due to the small sample size, this cannot contradict the well-known therapeutic benefit of corticosteroids in prolonging ambulation, and comparison of data regarding ambulation between Austria and Germany across all age groups was not significant (p = 0.097)^2,13–15^.

## Conclusion

In conclusion, until today, DMD is still not diagnosed early enough, and there is yet a considerable delay between onset of first symptoms and diagnosis, consecutively leading to delayed genetic counselling, thereby problems in family planning, late implementation of standards of care and tardy initiation of treatment. Therefore, we would strongly advise to thoroughly look for early signs of cognitive, verbal and motor delay in boys at pediatric routine check-ups around age 2 (similar to U7 at 21–24 months of age in Germany and 6th MKP in Austria at 22–26 months of age), and test for hyperCKemia.

## Supplementary Information

Supplementary Information.

## Data Availability

The datasets used and/or analysed during the current study are available from the corresponding author on reasonable request.
