# Supplementary material for: Time to diagnosis of Duchenne muscular dystrophy in Austria and Germany
Source: Sci Rep. 2023 Jan 5;13:179. doi: 10.1038/s41598-022-27289-2 (PMC9814243; doi:10.1038/s41598-022-27289-2)
Supplement: Supplementary file 1 — Supplementary Information. [file 41598_2022_27289_MOESM1_ESM.docx]

***Appendix***

Questionnaire on level of caregiver’s stress

We want to ask for your opinion on the following statements in relation to your current situation as your son’s caregiver. The statements refer to any kind of assistance you deliver, e.g. support, care or nurture.

|  |  | Strongly agree | Agree | Partly agree | Disagree | Strongly disagree | Not sure |
| --- | --- | --- | --- | --- | --- | --- | --- |
| 1 | I frequently feel physically exhausted |  |  |  |  |  |  |
| 2 | From time to time I have the desire to "break free" of my situation |  |  |  |  |  |  |
| 3 | My health is affected by the assistance I deliver as my son’s caregiver |  |  |  |  |  |  |
| 4 | The assistance I deliver as my son’s caregiver requires a lot of my own strength |  |  |  |  |  |  |
| 5 | Due to the assistance I deliver as my son’s caregiver, my relationship with other family members, relatives, and friends becomes adversely affected |  |  |  |  |  |  |
